# Evaluation of the Effect of a Growing up Milk Lite vs. Cow’s Milk on Diet Quality and Dietary Intakes in Early Childhood: The Growing up Milk Lite (GUMLi) Randomised Controlled Trial

**DOI:** 10.3390/nu11010203

**Published:** 2019-01-20

**Authors:** Amy L. Lovell, Tania Milne, Yannan Jiang, Rachel X. Chen, Cameron C. Grant, Clare R. Wall

**Affiliations:** 1 Discipline of Nutrition and Dietetics, Faculty of Medical and Health Sciences, University of Auckland, Auckland 1142, New Zealand; c.wall@auckland.ac.nz; 2 Faculty of Medical and Health Sciences, University of Auckland, Auckland 1142, New Zealand; t.milne@auckland.ac.nz; 3 Department of Statistics, Faculty of Science, University of Auckland, Auckland 1142, New Zealand; y.jiang@auckland.ac.nz (Y.J.); rachel.chen@auckland.ac.nz (R.X.C.); 4 Department of Paediatrics: Child and Youth Health, University of Auckland, Grafton 1023, New Zealand; cc.grant@auckland.ac.nz; 5 Centre for Longitudinal Research He Ara ki Mua, University of Auckland, Auckland 1743, New Zealand; 6 General Paediatrics, Starship Children’s Hospital, Auckland District Health Board, Auckland, Auckland 1142, New Zealand

**Keywords:** diet quality, PANDiet index, early childhood, nutritional adequacy, nutrient intake quality, growing up milk

## Abstract

Summary scores provide an alternative approach to measuring dietary quality. The Growing Up Milk-Lite (GUMLi) Trial was a multi-centre, double-blinded, randomised controlled trial of children randomised to receive a reduced protein GUM (GUMLi) or unfortified cow’s milk (CM). In a secondary analysis of the GUMLi Trial, we used the Probability of Adequate Nutrient Intake (PANDiet) to determine the nutritional adequacy of the diets of participating children living in Auckland. The PANDiet was adapted to the New Zealand Nutrient Reference Values and data from four 24 h Recalls (24HR) collected at months 7, 8, 10, and 11 post-randomisation were used. Differences between randomised groups (GUMLi vs. CM) of the PANDiet and its components were made. Eighty-three Auckland participants were included in the study (GUMLi *n* = 41 vs. CM *n* = 42). Total PANDiet scores were significantly higher in the GUMLi group (*p* < 0.001), indicating better overall nutrient adequacy and diet quality. Dietary intakes of children in both groups met the recommendations for fat, total carbohydrates and most micronutrients; however, protein intakes exceeded recommendations. Consumption of GUMLi was associated with higher nutritional adequacy, with an increased likelihood of meeting nutrient requirements; however, the impact of the family diet and GUMLi on dietary diversity requires further evaluation.

## 1. Introduction

Early food habits, practices, and dietary patterns develop rapidly within the first two years of life [1,2]; with evidence that diet quality may decline as children age [3]. Evaluating diet quality in paediatric populations is of increasing interest, however, due to a paucity of evidence-based dietary guidelines for children under two, combining these multidimensional behaviours into a single meaningful measure remains a challenge [4].

Diet quality can be determined using nutrient, food, or food and nutrient-based indices [5]. Index scores are determined ‘*a priori*’, using dietary guidelines, recommended nutrient intakes, or current nutrition knowledge of optimal dietary patterns [6,7,8,9]. The resulting numeric representation of dietary quality or nutrient adequacy can be used as a nutritional benchmark in identifying relationships between the whole-of-diet and later health [6,7,10,11]. Nutrient-based measures of diet quality reflect adequacy of nutrient intake, however, require detailed dietary assessment, additional analyses and statistical modelling before a final score is calculated [5,6]. In contrast, food-based indices provide an indirect measure of nutrient and non-nutrient interactions, where a score is easily calculated based on awarding points for fulfilling certain criteria [5,6]. The Probability of Adequate Nutrient Intake (PANDiet) score is a complete, nutrient-based diet quality index, employing probabilistic calculations of nutrient adequacy [12]. The index has been evaluated in French [12], US [12] adult populations and a UK [13] paediatric population and has shown to be a useful tool in assessing diet quality at the population level [12].

There is limited research on the contribution of milk to the diets of children under two [14,15,16], specifically, whether Growing Up Milks (GUM) provide a nutritional advantage compared to standard cow’s milk (CM) [17]. Simulation data have shown that replacing CM with GUM resulted in protein intakes more in line with recommendations, reduced saturated fatty acid (SFA) intake and increased likelihood of adequate intakes of vitamin D and iron [17,18]. We aimed to evaluate the dietary quality of the Auckland children participating in the GUMLi Trial aged 18- to 23-months, using an adapted PANDiet index and determine nutritional adequacy according to intervention allocation.

## 2. Materials and Methods

### 2.1. Study Design and Participants

This is a secondary analysis of data collected as part of the GUMLi trial. Briefly, the GUMLi trial was a multi-centre, double blinded, randomised controlled trial performed in Auckland, New Zealand (*n* = 108) and Brisbane, Australia (*n* = 52) from 2015 to 2017. One hundred and sixty healthy children aged one were randomised 1:1 to receive unfortified cow’s milk (CM) or a reduced protein GUM (GUMLi), fortified with iron, vitamin D, pre- and probiotics (Danone Pty Ltd., Auckland, New Zealand) until the age of two. GUMLi had a reduced energy and protein content compared to commercial GUM on the market, 60 kcal/100 mL vs. 71 kcal/100 mL and 1.7 g/100 mL protein vs. 2.2 g/100 mL. An energy-matched, non-fortified cow’s milk was used as an active control, with a protein content of 3.1 g/100 mL. The primary trial outcome evaluated the effect of consuming GUMLi versus unfortified CM as part of a whole diet for 12-months on body composition at two years of age [19]. Secondary outcomes included dietary intake (food frequency questionnaire or 24 h), micronutrient status, and cognitive development.

The study received ethical approval from the Health and Disability Ethics Committee of the Ministry of Health, New Zealand (14/NTB/152), and the University of Queensland Medical Research Ethics Committee, Brisbane, Australia (2014001318). The GUMLi Trial was registered with the Australian New Zealand Clinical Trials Registry, reference number: ACTRN12614000918628. Written informed consent was obtained from all participants. At month six post-randomisation, primary caregivers were invited to complete four record-assisted twenty-four-hour recalls (24 h). Of the 108 Auckland participants, 83 (77%) completed four 24 h. Four (4%) opted out of the study (but continued with the main trial), eleven (10 %) withdrew from the main trial and nine (8%) took part, but did not complete four 24 h. Only 14 (27%) participants from Brisbane completed four 24 h, therefore, the decision was made not to include them in the analysis.

### 2.2. Dietary Intakes

A dietitian collected dietary data over the phone using record-assisted 24HRs between months 6 to 11 post-randomisation, according to a standardised procedure [20]. Four 24 h were collected per participant on randomly allocated days (three weekdays and one weekend day). The record-assisted 24HR differed from standard 24HRs, as caregivers recoded their child’s intake over the pre-defined 24-h period preceding the phone call. This methodology was used in a pilot validation study for the New Zealand Children’s Nutrition Survey [21] and the Australian Children’s Nutrition and Physical Activity Survey (CNPAS) [22,23]. A ‘Foods fed by other adults’ form, adapted from the Feeding Infants and Toddlers study (FITS) [24,25] was used to record intake if the child was in the care of another adult i.e., day-care. Use of dietary supplements, homemade recipes, and portion sizes (household measures or gram weight) were recorded. A food model booklet, reproduced with permission from CNPAS was used to assist with describing serving sizes [22,23]. Breastfeeding was recorded as time (minutes) and quantity estimated using a conversion factor of 10 mL/min, max 10 min [26,27]. All 24HR were double-checked to identify mistakes, missing foods, or clarify recipes. A dietetics student entered the data into Foodworks^®^ (version 9, Xyris Software, Pty Ltd., Australia) and checked for completeness. Nutritional data were derived from the FOODfiles 2016 database [28] and nutritional profiles of commercial toddler foods sourced from companies or nutrient information panels.

### 2.3. Assessment of Nutrient Intakes with Nutrient Reference Values

Nutrient intakes were compared to the Australian and New Zealand Nutrient Reference Values (NRVs) [29]. Prevalence of inadequate intakes were assessed using the cut-off point method for nutrients with an Estimated Average Requirement (EAR) value [30]. This method has previously been shown to produce realistic estimates of the prevalence of inadequate dietary intakes [30]. The EAR, derived by the Institute of Medicine (IoM) was used for vitamin D (10 µg/day) [31].

### 2.4. Assessment of Diet Quality Using the PANDiet Score

The development and design of the PANDiet score has been reported in detail elsewhere [12,32]. Briefly, the PANDiet provides a measure of diet quality through the probability of having adequate nutrient intake, ranging from 0–100, where the higher the score, the better the diet quality and nutrient adequacy [12,32]. The PANDiet is an average of the Adequacy and Moderation sub-scores, which rely on the calculation of probability of adequacy for 25 nutrients and consider duration of dietary assessment, day-to-day variability, nutrient reference values, inter-variability of intake, and mean nutrient intakes [12,32]. The Adequacy sub-score calculates the probability that usual nutrient intake is above a reference value and the Moderation sub-score calculates the probability that the usual nutrient intake meets requirements and does not exceed a reference value [12,33]. Using the original methods [12], the PANDiet score calculation for protein and micronutrients was adjusted using the Australian and New Zealand NRVs and inter-variability for children one- to three years of age [29]. There are no recommendations for total fat, poly-unsaturated fatty acids (PUFA) and carbohydrate in children under two. Therefore, as seen in Verger et al. [32], we used the reference values set by the European Food Safety Authority (EFSA) [34], Nordic recommendations for SFA and non-milk extrinsic sugars (NMES) [35] and the IoM upper limit for protein [36]. The risk of excessive intakes were assessed using a penalty value system [12], using the upper limit as a reference [29] (Table S1). 

Participants were classified according to their randomisation into the trial and allocation to receive GUMLi or CM. The trial analysis was conducted based on the assumption that the PANDiet index was suitable to use as an outcome measure and the difference between randomised groups, if observed, would indicate an effect of the intervention.

### 2.5. Statistical Analysis

A sample size of 64 participants in each arm is required to have 80% power at 5% significant level (two-sided) to detect a 0.5 SD difference in body fat percent (primary outcome) between the two arms at the end of the 12-month intervention. Statistical analyses were performed using SAS version 9.4 (SAS Institute Inc., Cary, NC, USA). Baseline characteristics were summarised by treatment group (GUMLi vs. CM) using descriptive statistics. Continuous variables were reported as mean and standard deviation (SD) and categorical variables described as frequencies and percentages. The characteristics of the Auckland sub-group included in this study (*N* = 83) were compared to those in the Auckland cohort who did not participate (*N* = 25). Chi-Square test or Fisher’s Exact test were used for categorical variables, and the Kruskal-Wallis test or two-sample *t*-test was used for continuous variables. The impact of the intervention on energy and nutrient intakes were evaluated at each 24HR time point (month 7, 8, 10, and 11 post-randomisation), using random effect mixed models with an autoregressive covariance structure on repeated measures. The fixed effects model included participant sex, treatment group, time point and its interaction with the treatment group. Model-adjusted mean differences between nutrient intakes of both groups and 95% confidence intervals (95% CI) were reported at each time point, with associated *p*-values. The impact of the intervention on the overall PANDiet score, sub-scores and components using all 24 h data were evaluated using linear regression models adjusting for sex. Model-adjusted mean differences between two groups were estimated and tested. All statistical tests were two-sided with a statistical significance of *p* < 0.05. As a secondary analysis, missing data was not imputed and no adjustment for multiple comparisons were considered.

## 3. Results

One hundred and eight Auckland children participated in the main GUMLi trial. Of these, 83 (77%) were included in this sub-study, with no significant differences between GUMLi and CM groups for any baseline characteristics (Table 1), therefore, it was assumed that any differences in PANDiet scores would be attributed to the intervention milk. No statistical differences were observed between the Auckland participants included in the analysis (*N* = 83) and those excluded (*N* = 25), except for maternal educational attainment (80% vs. 60%; *p* = 0.047) (Table S2). GUMLi and CM composition are presented in Table S3. Both milks were energy-matched per 100 mL, however compared to CM, GUMLi was lower in SFA and protein, with higher carbohydrate and dietary fibre, and nutritionally significant amounts of iron and vitamin D (cholecalciferol).

### 3.1. Evaluation of Nutrient Intakes

Mean (SD) daily nutrient intakes at the four 24HR time points are displayed in Table 2, according to GUMLi or CM group. For the purpose of table length, only nutrients with significant relationships at any time point are displayed. A full table is presented in Table S4. There were no differences between groups at any time point for energy, sodium, PUFA, vitamin A, vitamin B-6, folate, magnesium, and selenium. Children in the GUMLi group had significantly higher intakes of vitamin C and iron across all time points, and children in the CM group had significant higher intakes of riboflavin and potassium. 

**Figure 1:**
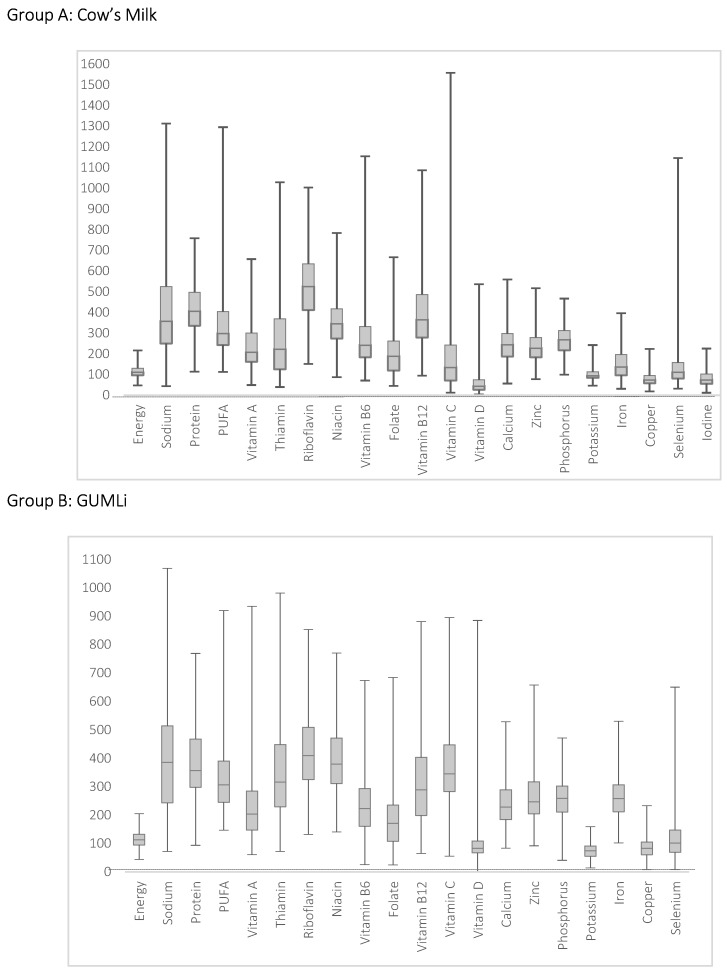
Intake of energy and nutrients as a percentage of New Zealand reference values (28) in 18- to 23-month-old children from the Auckland cohort participating in the GUMLi trial (median (—), interquartile range (box; 25th and 75th percentiles), minimum and maximum value). GUMLi = Growing Up Milk—Lite.

Compared with New Zealand NRVs [29], intakes of most nutrients were adequate, i.e., median intake (average all four 24 h) ≥ nutrient reference value across both groups Figure 1. Nutrients with median intakes below reference values in both groups were vitamin D, potassium, copper, and iodine.

### 3.2. PANDiet Scores According to Intake of GUMLi or CM

Mean PANDiet score, sub-scores and individual components are displayed in Table 3. After adjusting for sex, children in the GUMLi group had significantly higher PANDiet scores and Adequacy scores compared to the CM group (adjusted mean difference +3.11 and +4.17, respectively). There was no difference in the Moderation sub score and energy intake between groups. Of note, the Adequacy sub-score was 2.5 and 2.6 times greater than the Moderation sub-score in the GUMLi and CM group, respectively, indicating poor adherence to the recommendations for avoiding excessive nutrient intakes.

There were no differences in component of the Moderation sub-score, except for total fat and total carbohydrates, where the CM group had a higher probability of avoiding excessive total fat intake and the GUMLi group had a higher probability of avoiding excessive total carbohydrate intake. The GUMLi group tended to have higher probability of avoiding excessive protein intake (not significant). The mean probabilities for avoiding excessive intakes were low for sodium (≤0.03), SFA (≤0.10) and NMES (≤0.23) in this population. There were no differences between groups in components of the Adequacy sub-score, except for total fat, thiamin, vitamin C, vitamin D, iron, and iodine where the GUMLi group had a higher probability of having adequate intakes for these nutrients, and vitamin B12 where the CM group had a higher probability of having an adequate intake.

## 4. Discussion

Using the PANDiet index, we have evaluated the diet quality and nutritional adequacy of 18- to−23-month-old Auckland children participating in the GUMLi Trial, according to GUMLi or CM consumption. This is the first study to use data from a randomised controlled trial to measure the impact of a dietary intervention, such as GUM on diet quality using a nutrient-based index, such as the PANDiet score. Total PANDiet scores were significantly higher in the GUMLi group, indicating better overall nutrient adequacy and diet quality. Nutrient intakes of children in both groups met recommendations for fat, total carbohydrates and most micronutrients; however, protein sodium, NMES, and SFA intakes exceeded recommendations. Whilst average total energy intakes were similar, the children consuming GUMLi had higher probabilities of having adequate intakes of vitamin C, vitamin D and iron, and were less likely to have insufficient intakes of vitamin D. Further analysis of food group consumption, adherence to dietary guidelines, or nutrient densities would elucidate whether the GUMLi intervention had an impact on dietary diversity, as an inverse relationship between dietary diversity and formula intake has previously been reported in 12- to 16-month-old Australian children [16].

### 4.1. Diet Quality and PANDiet Scores According to GUMLi or CM Allocation

GUM has been shown to improve intakes of iron, vitamin C, vitamin D, and PUFA’s during the dietary transition from a milk-based intake to a ‘family diet’ in cross-sectional, observational studies [14,15,16,37]. The PANDiet has previously been evaluated in 12- to 18-month-old-children in the U.K. according to GUM or commercial infant foods (CIF) consumption [32]. Consuming GUM was associated with greater nutritional adequacy with a mean PANDiet score of 74.1 compared to children who did not consume GUM or CIF (difference of +7.2 points) [32]. A much smaller difference of +2.78 was observed in our sample, where consuming GUMLi was associated with greater nutritional adequacy. More recently, the difference in PANDiet scores for ‘at risk children with Diabetic mothers’ and ‘not at risk’ children in the BABYDIET study was similar at +2.4 points (65.9 and 68.3, respectively) [13].

It is important to note the effect of differences in national nutrient recommendations on PANDiet calculations and resultant scores. In the present study, the PANDiet score calculation was adjusted according to Australia and New Zealand NRVs where available [29], and if not, the reference values determined by Verger et al. [32] who used nutrient recommendations for UK children 12- to 36-months-of-age [34,35,38]. The greatest variation in recommendations were for selenium and folate, where the New Zealand NRVs are 1.7–2.4 times higher than the UK, (folate: 120 µg vs. 50 µg and selenium: 20 µg vs. 11.5 µg). The probability of adequate would be higher than the current calculation if we used the UK recommendations in our sub-score calculation. As all probabilities of adequacy are equally weighted, higher component scores will contribute to a higher total Adequacy sub-score and resultant PANDiet score [12].

In this population, the quality of both fat and carbohydrates are of concern. Children had low probabilities for avoiding excessive intakes for SFA (≤0.10) and low probabilities for having adequate PUFA intakes (<0.20). An altered ratio of total and SFA has been described in two other studies, and is an important consideration, given the role of PUFA’s in cognitive and visual development [37,39]. At each time point in the study, NMES exceeded the recommendations (>11% EI). Similar intakes were observed in a nationally representative sample of one- to four-year-old Irish children, where mean NMES intakes exceeded recommendations at 12% energy intake (EI) and increased with age [40].

### 4.2. Strengths and Limitations

The PANDiet provides an accurate measure of diet quality at an individual and population level through assessing global nutrient adequacy, and is strengthened by the use of a probabilistic calculation of nutrient adequacy, as previously described [12]. The index was designed to be as exhaustive as possible, and describes the role that different foods/food groups have in contributing to diet quality, at the nutrient level [12]. Our analysis is strengthened by the use of New Zealand NRVs to assess nutrient adequacy in the New Zealand context, however, because of this, cross-national comparisons of the PANDiet score are limited. Previous studies have used large, observational cohorts, where each subject has one PANDiet score calculated at a single time point, using multiple measures of dietary assessment [12,32,33]. For the present study, dietary data were collected on one day per month, over four months; therefore, month-to-month variation was considered in the PANDiet calculation. Using a record-assisted 24HR, allowed inclusion of children in the care of other adults (i.e., at day care), however, the reliance on parent and other adult-reported measures may lead to an increase in misreporting or social desirability bias. Mother’s in our sample were older and highly educated, which may have affected total PANDiet scores. The ethnicity distribution in our sample was not considered representative of the Auckland population; therefore, no differences between ethnicities were evaluated. The validity of the PANDiet index was not evaluated for RCT data, therefore, further evaluation of the PANDiet in a larger, more representative cohort of New Zealand children under two is recommended to determine whether the PANDiet has predictive validity with respect to longitudinal health outcomes.

## 5. Conclusions

The consumption of GUMLi was associated with higher nutritional adequacy of the diets of children 18- to 23-months-of-age defined by PANDiet score, with increased likelihood of meeting nutrient requirements. However, consumption of GUMLi did not guarantee 100% nutrient adequacy. GUMLi consumers still had excessive protein intakes, but were more likely to have carbohydrate and SFA intakes that were in line with recommendations and improved iron and vitamin D intakes. Although GUMLi had a positive effect on index scores, consumption toward the latter half of the second year of life may not have the same impact as during early childhood as previously reported in younger children according to GUM consumption [32]. This may be because in the latter part of the second year of life, children are more likely to be following a family diet of varying quality, with a reduced reliance on fortified milks. Suggesting that other dietary strategies to promote a healthy diet through optimising nutrient intake could also result in more favourable dietary intake profiles, rather than solely concentrating on milk [41], however, further research is required on the consequences of consuming GUMLi on overall dietary diversity.

## Figures and Tables

**Table 1:** Child and maternal characteristics of the Auckland cohort (*N* = 83) included in the PANDiet cohort.

Baseline Demographics	Study Group	*p*-Value *	
	Intervention (*N* = 41) *n* (%)	Control (*N* = 42) *n* (%)	
Child’s sex			0.062
Boy	19 (46)	28 (67)	
Girl	22 (54)	14 (33)	
Other children in the family			0.222
No	16 (39)	22 (52)	
Yes	25 (61)	20 (48)	
Day care attendance			0.893
No	25 (61)	25 (60)	
Yes	16 (39)	17 (40)	
Breastfed at baseline			0.415
No	27 (66)	24 (57)	
Yes	14 (34)	18 (43)	
Mother’s Ethnicity			0.903
Māori	8 (20)	6 (14)	
Pacific	0 (0)	1 (2)	
Asian	3 (7)	2 (5)	
European	23 (56)	26 (62)	
Other	7 (17)	7 (17)	
Mother’s Age, years (mean ± SD)	32 ± 5	32 ± 4	0.874
Mother’s BMI, kgm^2^ (mean ± SD)	26 ± 5	27 ± 6	0.916
Mother’s Highest Level of Education			0.589
No school qualifications	0(0)	0(0)	
Primary	2 (5)	0 (0)	
Secondary	5 (12)	7 (17)	
Tertiary	33 (80)	33 (79)	
Other	1 (2)	2 (5)	
Mother’s Employment Status			0.082
Full-time caregiver	14 (34)	15 (36)	
Full-time paid employment	5 (12)	13 (31)	
Part-time paid employment	14 (34)	13 (31)	
Receiving a benefit	1 (2)	0 (0)	
Unemployed, no benefit	3 (7)	0 (0)	
Other	4 (10)	1 (2)	
Smoking			
Current smoking	1 (2)	1 (2)	1.000
Smoking before pregnancy	5 (12)	2 (5)	0.432
Smoking during pregnancy	1 (2)	0 (0)	0.494

* Unadjusted *p*-values, Chi-square test or Fisher’s Exact test is used to test the difference between groups for categorical variables; the Kruskal-Wallis test or two-sample *t*-test is used to compare the medians/means between groups for continuous variables.

**Table 2:** Nutrient intake among Auckland children (*N* = 83) from 18 and 23 months of age (month 7–11 post randomisation) ^1,2^.

	Usual Intake Values		Adjusted Difference (95%CI)	*p* *
Nutrients	Intervention (*N* = 41) Mean (SD)	Control (*N* = 42) Mean (SD)		
Energy (kcal)				
Month 07	1135.92 (294.19)	1122.34 (187.51)	36.61 (−93.18, 166.41)	0.579
Month 08	1114.07 (277.52)	1246.07 (378.83)	−108.96 (−238.76, 20.84)	0.100
Month 10	1128.31 (383.78)	1068.61 (291.44)	82.74 (−47.05, 212.54)	0.210
Month 11	1190.24 (288.14)	1118.93 (283.21)	94.34 (−35.45, 224.14)	0.154
Carbohydrate (g)				
Month 07	142.45 (40.53)	127.44 (36.36)	18.26 (−0.25, 36.76)	0.053
Month 08	138.50 (38.12)	144.65 (56.59)	−2.90 (−21.41, 15.61)	0.758
Month 10	138.25 (45.72)	123.41 (41.01)	18.09 (−0.42, 36.59)	0.055
Month 11	145.81 (41.01)	126.61 (43.06)	22.45 (3.94, 40.96)	0.018 *
Total fat (g)				
Month 07	38.49 (13.70)	43.91 (11.34)	−4.69 (−11.04, 1.65)	0.146
Month 08	37.76 (14.79)	46.21 (16.22)	−7.72 (−14.07, −1.37)	0.017 *
Month 10	39.53 (17.61)	40.34 (13.50)	−0.08 (−6.42, 6.27)	0.981
Month 11	43.99 (15.81)	43.75 (13.59)	0.97 (−5.38, 7.32)	0.764
Saturated fat (g)				
Month 07	18.98 (7.37)	21.16 (5.98)	−1.96 (−5.27, 1.34)	0.243
Month 08	18.11 (7.41)	22.16 (8.16)	−3.83 (−7.14, −0.53)	0.023 *
Month 10	19.46 (8.88)	19.73 (7.77)	−0.05 (−3.35, 3.26)	0.977
Month 11	20.93 (8.11)	21.00 (6.84)	0.15 (−3.16, 3.45)	0.930
NMES (g)				
Month 07	45.46 (18.22)	42.02 (17.88)	4.33 (−5.27, 13.93)	0.375
Month 08	45.90 (19.00)	49.01 (30.16)	−2.22 (−11.83, 7.38)	0.649
Month 10	40.13 (23.17)	39.00 (19.24)	2.03 (−7.58, 11.63)	0.678
Month 11	48.53 (25.02)	39.29 (21.47)	10.14 (0.54, 19.74)	0.039 *
Protein (g)				
Month 07	46.07 (17.15)	50.13 (10.13)	−3.26 (−9.65, 3.13)	0.316
Month 08	46.09 (14.01)	56.47 (17.08)	−9.58 (−15.97, −3.19)	0.004 *
Month 10	46.45 (18.34)	44.33 (12.51)	2.92 (−3.47, 9.31)	0.369
Month 11	44.59 (14.36)	47.48 (13.13)	−2.09 (−8.48, 4.30)	0.520
Thiamin (mg)				
Month 07	1.50 (0.63)	1.19 (0.84)	0.34 (0.03, 0.64)	0.030 *
Month 08	1.54 (0.56)	1.29 (0.72)	0.28 (−0.02, 0.59)	0.069
Month 10	1.35 (0.70)	1.03 (0.82)	0.36 (0.05, 0.66)	0.022 *
Month 11	1.36 (0.68)	0.99 (0.64)	0.40 (0.10, 0.71)	0.010 *
Riboflavin (mg)				
Month 07	1.82 (0.64)	2.12 (0.64)	−0.29 (−0.56, −0.02)	0.037 *
Month 08	1.71 (0.54)	2.30 (0.77)	−0.58 (−0.85, −0.30)	<0.0001 *
Month 10	1.66 (0.50)	2.07 (0.67)	−0.39 (−0.66, −0.11)	0.006 *
Month 11	1.63 (0.61)	2.11 (0.57)	−0.47 (−0.74, −0.20)	0.001 *
Niacin (mg)				
Month 07	19.97 (7.25)	17.79 (4.64)	2.49 (−0.12, 5.09)	0.061
Month 08	20.63 (5.18)	20.09 (7.30)	0.85 (−1.75, 3.45)	0.521
Month 10	19.34 (6.64)	15.80 (5.87)	3.84 (1.24, 6.45)	0.004 *
Month 11	19.09 (5.31)	17.28 (5.39)	2.11 (−0.49, 4.71)	0.112
Vitamin B12 (µg)				
Month 07	2.36 (1.12)	2.78 (1.09)	−0.41 (−0.91, 0.09)	0.108
Month 08	2.25 (1.30)	3.17 (1.55)	−0.91 (−1.41, −0.41)	0.0004 *
Month 10	2.14 (0.94)	2.57 (0.93)	−0.42 (−0.92, 0.07)	0.095
Month 11	2.03 (0.91)	2.58 (1.15)	−0.55 (−1.05, −0.05)	0.031 *
Vitamin C (mg)				
Month 07	104.00 (44.39)	45.38 (37.36)	57.38 (35.76, 79.01)	<0.0001 *
Month 08	99.95 (39.44)	50.22 (54.71)	48.48 (26.86, 70.11)	<0.0001 *
Month 10	92.84 (34.62)	50.54 (65.97)	41.05 (19.43, 62.68)	0.0002 *
Month 11	92.51 (48.54)	58.80 (61.49)	32.47 (10.85, 54.10)	0.003 *
Vitamin D (µg)				
Month 07	6.02 (6.57)	3.23 (3.18)	2.80 (1.07, 4.53)	0.002 *
Month 08	4.73 (2.70)	3.59 (4.00)	1.16 (−0.57, 2.89)	0.188
Month 10	5.17 (2.76)	2.92 (2.49)	2.27 (0.54, 4.00)	0.011 *
Month 11	4.86 (3.44)	3.73 (4.75)	1.15 (−0.58, 2.88)	0.194
Calcium (mg)				
Month 07	901.26 (268.15)	898.06 (287.37)	8.15 (−113.76, 130.06)	0.895
Month 08	808.31 (257.94)	943.49 (314.09)	−130.24 (−252.15, −8.33)	0.036 *
Month 10	899.34 (284.54)	836.03 (251.58)	68.25 (−53.65, 190.16)	0.271
Month 11	830.41 (284.29)	891.05 (280.79)	−55.70 (−177.60, 66.21)	0.369
Zinc (mg)				
Month 07	6.75 (2.76)	6.11 (1.51)	0.71 (−0.22, 1.64)	0.133
Month 08	6.64 (2.24)	6.85 (2.65)	−0.13 (−1.07, 0.80)	0.776
Month 10	6.44 (2.45)	5.37 (1.74)	1.14 (0.21, 2.07)	0.017 *
Month 11	6.42 (1.77)	5.45 (1.69)	1.04 (0.11, 1.97)	0.029 *
Phosphorus (mg)				
Month 07	1023.06 (284.32)	1004.05 (202.02)	33.99 (−83.88, 151.87)	0.571
Month 08	966.96 (257.85)	1106.43 (293.65)	−124.49 (−242.36, −6.62)	0.039 *
Month 10	984.67 (335.64)	930.98 (252.54)	68.68 (−49.20, 186.55)	0.252
Month 11	989.53 (278.71)	988.18 (262.19)	16.33 (−101.54, 134.20)	0.785
Potassium (mg)				
Month 07	1666.69 (703.04)	1962.08 (433.28)	−283.10 (−528.37, −37.83)	0.024 *
Month 08	1537.51 (481.42)	2232.75 (761.17)	−682.95 (−928.21, −437.68)	<0.0001 *
Month 10	1406.79 (493.64)	1861.05 (503.26)	−441.97 (−687.24, −196.70)	0.001 *
Month 11	1512.12 (526.22)	1987.09 (511.40)	−462.67 (−707.94, −217.40)	0.000 *
Iron (mg)				
Month 07	10.62 (3.36)	6.23 (2.82)	4.58 (3.31, 5.85)	<0.0001 *
Month 08	10.80 (2.96)	6.90 (2.75)	4.10 (2.83, 5.37)	<0.0001 *
Month 10	9.83 (2.89)	5.64 (3.07)	4.38 (3.11, 5.65)	<0.0001 *
Month 11	10.26 (3.24)	5.24 (2.35)	5.21 (3.93, 6.48)	<0.0001 *
Copper (mg)				
Month 07	0.62 (0.32)	0.6 (0.24)	0.04 (−0.08, 0.15)	0.524
Month 08	0.6 (0.26)	0.68 (0.35)	−0.06 (−0.18, 0.05)	0.255
Month 10	0.63 (0.28)	0.5 (0.21)	0.15 (0.04, 0.26)	0.010 *
Month 11	0.6 (0.18)	0.53 (0.18)	0.09 (−0.02, 0.2)	0.115
Iodine (µg)				
Month 07	64.08 (23.15)	52.65 (24.78)	11.80 (0.49, 23.11)	0.041 *
Month 08	63.58 (29.84)	55.72 (21.88)	8.22 (−3.09, 19.54)	0.154
Month 10	60.53 (27.92)	53.13 (26.95)	7.77 (−3.55, 19.08)	0.178
Month 11	65.92 (30.56)	53.53 (21.06)	12.76 (1.45, 24.07)	0.027

* *p* < 0.05. ^1^ Repeated measures mixed model with an autoregressive covariance structure, adjusting for sex. ^2^ Only nutrients with significant relationships at any of the four time points are displayed.

**Table 3:** PANDiet scores, sub-scores, and individual components, among Auckland children (*N* = 83) from 18 and 23 months of age (month 7–11 post randomisation) ^1,2^.

Score	Intervention (*N* = 41)	Control (*N* = 42)	Adjusted Difference (95% CI)	*p*-Value *
	Mean (SD)	Mean (SD)		
PANDiet ^3^	52.9 (3.07)	50.12 (3.97)	3.11 (1.56, 4.67)	0.0001 *
Moderation sub-score	29.82 (6.47)	27.77 (6.58)	2.06 (−0.87, 4.99)	0.1660
Protein	0.41 (0.50)	0.33 (0.48)	0.08 (−0.14, 0.30)	0.4747
Total Fat	0.10 (0.30)	0.48 (0.51)	−0.40 (−0.59, −0.22)	<0001 *
Total Carbohydrate	0.85 (0.36)	0.55 (0.50)	0.33 (0.14, 0.53)	0.0011 *
SFA	0.10 (0.16)	0.06 (0.12)	0.05 (−0.01, 0.11)	0.1231
NMES	0.16 (0.22)	0.23 (0.28)	−0.08 (−0.19, 0.03)	0.1699
Sodium	0.03 (0.14)	0.02 (0.04)	0.01 (−0.03, 0.06)	0.5832
Adequacy sub-score	75.98 (4.98)	72.46 (5.88)	4.17 (1.82, 6.51)	0.0007 *
Protein	0.99 (0.03)	1.00 (0.001)	−0.01 (−0.01, 0.002)	0.1697
Total Carbohydrate	0.98 (0.16)	1.00 (0.00)	−0.02 (−0.07, 0.03)	0.4293
Total Fat	1.00 (0.00)	0.86 (0.35)	0.14 (0.03, 0.26)	0.0140 *
PUFA	0.15 (0.19)	0.18 (0.23)	−0.01 (−0.10, 0.08)	0.7975
Vitamin A	0.96 (0.08)	0.98 (0.02)	−0.02 (−0.05, 0.001)	0.0560
Thiamin	1.00 (0.005)	0.94 (0.09)	0.06 (0.03, 0.09)	0.0001 *
Riboflavin	1.00 (0.001)	1.00 (0.0003)	−0.0003 (−0.001, 0.00)	0.0853
Niacin	1.00 (0.00001)	1.00 (0.0005)	0.0001 (0.00, 0.0002)	0.1819
Vitamin B6	0.00 (0.00)	0.00 (0.00)	0.00 (0.00,0.00)	0.9775
Folate	0.89 (0.18)	0.91 (0.21)	−0.01 (−0.10, 0.07)	0.7717
Vitamin B12	0.99 (0.02)	1.00 (0.01)	−0.01 (−0.02, −0.003)	0.0081 *
Vitamin C	1.00 (0.01)	0.71 (0.32)	0.30 (0.20, 0.40)	<0001 *
Vitamin D	0.43 (0.34)	0.19 (0.31)	0.25 (0.10, 0.39)	0.0011 *
Calcium	0.99 (0.02)	1.00 (0.02)	−0.002 (−0.01, 0.01)	0.7227
Magnesium	0.99 (0.02)	1.00 (0.004)	−0.005 (−0.01, 0.002)	0.1826
Zinc	1.00 (0.003)	0.99 (0.02)	0.01 (−0.0002, 0.01)	0.0585
Phosphorus	1.00 (0.01)	1.00 (0.002)	−0.001 (−0.003, 0.0005)	0.1323
Potassium	1.00 (0.002)	1.00 (1E−6)	−0.0003 (−0.001, 0.0002)	0.1998
Iron	1.00 (0.01)	0.78 (0.32)	0.25 (0.16, 0.35)	<0001 *
Copper	0.34 (0.30)	0.27 (0.27)	0.10 (−0.03, 0.23)	0.1203
Selenium	0.57 (0.37)	0.70 (0.28)	−0.09 (−0.23, 0.06)	0.2314
Iodine	0.46 (0.28)	0.29 (0.24)	0.18 (0.06, 0.30)	0.0035 *

NMES, Non-milk Extrinsic Sugars; PANDiet, The Probability of Adequate Nutrient Intake score; PUFA, Poly0unsaturated Fatty Acids; SFA, Saturated Fatty Acids. * *p* < 0.05. ^1^ Linear regression model, adjusting for sex. ^2^ All the PANDiet component scores range from 0 to 1, where 1 represents a 100% probability that the intake is adequate according to a reference value. ^3^ Combined data from all four 24HR were used to calculate the overall PANDiet score and adequacy and moderation sub-scores, which ranged from 0 to 100. The higher the score or sub-scores, the better the nutrient adequacy of the diet.

## Supplementary Materials

The following are available online at http://www.mdpi.com/2072-6643/11/1/203/s1, Table S1. PANDiet for Australian and New Zealand children aged from 18 to 23 months: components, reference values and inter-individual variability, Table S2. Nutritional composition of CM and GUMLi per 100 mL of prepared product, Table S3. Characteristics of the Auckland cohort included versus excluded in the PANDiet analysis, Table S4. Nutrient intake among Auckland children (*N* = 83) from 18 to 23 months of age (month 7–11 post randomisation).
