# Supplementary material for: Evaluation of the Effect of a Growing up Milk Lite vs. Cow’s Milk on Diet Quality and Dietary Intakes in Early Childhood: The Growing up Milk Lite (GUMLi) Randomised Controlled Trial
Source: Nutrients. 2019 Jan 20;11(1):203. doi: 10.3390/nu11010203 (PMC6356321; doi:10.3390/nu11010203)
Supplement: Supplementary file 1 [file nutrients-11-00203-s001.pdf]

Table S1. PANDiet for Australian and New Zealand children aged from 18 to 23 months: components, reference values, and inter-individual variability [1].

	Reference Value	Variability	Ref
**Moderation sub-score**			
Protein	3.5 g/kg	0%	
Total Fat	40% of EI	0%	
Total Carbohydrate	60% of EI	0%	
SFA	11 % of EI[#]	15%[#]	
NMES	11% of EI[#]	15%[#]	
Sodium	400 mg	15%	
**Adequacy sub-score**			
Protein	0.92 g/kg	12%	
Total Carbohydrate	45% of EI[#]	0%[#]	
Total Fat	35% of EI[#]	0%[#]	
PUFA	4.5 % of EI	15%[#]	
Vitamin A	210 µg	20%	
Thiamin	0.4 mg/1000 kcal	10%	
Riboflavin	0.4 mg	10%	
Niacin	5 mg/1000 kcal	15%	
Vitamin B_6	15 µg/g protein	10%	
Folate	120 µg	10%	
Vitamin B_{12}	0.7 µg	10%	
Vitamin C	25 mg	20%	
Vitamin D	5 µg	15%[#]	
Calcium	360 mg	15%	
Magnesium	5.0mg/kg	10%	
Zinc	2.5 mg	10%	
Phosphorus	380 mg	10%	
Potassium	200 mg	15%[#]	
Iron	4 mg	15%[#]	
Copper	0.7 mg	15%[#]	
Selenium	20 µg	10%	
Iodine	65 µg	20%	
**Tolerable Upper Intake Limits**			
Retinol (vitamin A)	600 µg		
Niacin	250 mg		
Vitamin B-6	15 mg		
Vitamin C	400 mg		
Vitamin D	80 µg		
Folate	300 µg		
Calcium	2, 500 mg		
Zinc	7 mg		
Phosphorus	3,000 mg		
Iron	20 mg		

[1] EI, Energy intake. Reference value refers to Australia and New Zealand nutritional recommendations including Estimated Average Requirement (EAR), adequate Intake (AI) or Acceptable Macronutrient Distribution Range, as appropriate (28).

#Data not available in New Zealand and Australian nutrient reference values. Values obtained from Verger et al. (31) for U.K. children (33,34).
† Reference value obtained from the Institute of Medicine (IoM) upper limit for protein in 1–3 year old children (35)

Table S2. Nutritional composition of CM and GUMLi per 100 mL of prepared product.

	Study Group	
	**Control CM[1]**	**Intervention GUML[1]**
Energy (kJ)	245.0	249.0
(kcal)	71.0	60.0
**Macronutrients**		
Protein (g)	3.1	1.7
Carbohydrate (g)	4.5	7.8
Total Fat (g)	3.6	1.9
Saturated (g)	1.9	1.3
Total n-3 long chain fatty acids (g) (DHA+EPA+DPA[2])	<0.002	0.04
Dietary Fiber[3]		
scGOS (g)	0.0	1.8
lcFOS (g)	0.0	0.2
**Micronutrients**		
Sodium (mg)	24.7	19.0
Calcium (mg)	96.7	116.0
Phosphorus (mg)	81.5	94.0
Magnesium (mg)	9.0	11.0
Non heme iron (mg)	0.0	1.3
Zinc (mg)	0.3	0.5
Iodine (μg)	2.4	9.1
Vitamin A (μg REs)	33.4	27.0
Cholecalciferol (μg)	0.1	1.2
dl-alpha-tocopherol (mg)	0.1	1.3
Thiamin (mg)	0.02	0.1
Riboflavbin (μg)	193.8	137.0
Vitamin B-6 (μg)	0.5	139.0
Vitamin B-12 (μg)	0.42	0.14
Niacin (μg)	0.1	1.0
Folic acid (μg)	0.5	20.0
Vitamin C (mg)	0.0	17.0

[1]Obtained from manufacturer (Danone), values based on average totals from three batches produced for use in the GUMLi trial.
[2]DHA: Docosahaexaenoic acid; EPA: Eicosapentaenoic acid; DPA: Docosapentaenoic acid.
[3]scGOS: Short-chain galacto-oligosaccharides; lcFOS: Long-chain fructo-oligosaccharides.

Table S3. Characteristics of the Auckland cohort included versus excluded in the PANDiet analysis.

	Auckland Participants		
	Included (N=83)	Excluded (N=25)	
**Baseline demographics**	**n (%)**	**n (%)**	**P***
**Child's sex**			0.765
Boy	47 (57)	15 (60)	
Girl	36 (43)	10 (40)	
**Other children in the family**			0.585
No	38 (46)	13 (52)	
Yes	45 (54)	12 (48)	
**Day care attendance**			0.464
No	50 (60)	13 (52)	
Yes	33 (40)	12 (48)	
**Breastfed at baseline**			0.522
No	51 (61)	13 (54)	
Yes	32 (39)	11 (46)	
Missing		1	
**Mother's Ethnicity**			0.196
Māori	14 (17)	1 (4)	
Pacific	1 (1)	1 (4)	
Asian	5 (6)	1 (4)	
European	49 (59)	20 (80)	
Other	14 (17)	2 (8)	
**Mother's Age, years** (mean ± SD)	32 (5)	30 (6)	0.102
**Mother's BMI, kgm²** (mean ± SD)	27 (6)	27 (5)	0.957
**Mother's Highest Level of Education**			**0.047**
No school qualifications	0 (0)	0 (0)	
Primary	2 (2)	0 (0)	
Secondary	12 (14)	10 (40)	
Tertiary	66 (80)	15 (60)	
Other	3 (4)	0 (0)	
**Mother's Employment Status**			0.136
Full-time caregiver	29 (35)	7 (28)	
Full-time paid employment	18 (22)	7 (28)	
Part-time paid employment	27 (33)	6 (24)	
Receiving a benefit	1 (1)	2 (8)	
Unemployed, not receiving a benefit	3 (4)	2 (8)	
Other	5 (6)	0 (0)	
Prefer not to answer	0 (0)	1 (4)	
**Smoking**			
Current smoking	2 (2)	0 (0)	1.000
Smoking before pregnancy	7 (8)	2 (8)	1.000
Smoking during pregnancy	1 (1)	1 (4)	0.411

*Chi-square test or Fisher's Exact test is used to test the difference between groups for categorical variables; the Kruskal-Wallis test or two-sample t-test is used to compare the medians/means between groups for continuous variables.

Table S4. Nutrient intake among Auckland children (N=83) from 18 and 23 months of age (month 7–11 post randomisation)[1,2].

Nutrients	Intervention (N=41) Mean (SD)	Control (N=42) Mean (SD)	Adjusted Difference (95% CI)	P*
**Energy, kcal**				
Month 07	1135.92 (294.19)	1122.34 (187.51)	36.61 (–93.18, 166.41)	0.579
Month 08	1114.07 (277.52)	1246.07 (378.83)	–108.96 (–238.76, 20.84)	0.100
Month 10	1128.31 (383.78)	1068.61 (291.44)	82.74 (–47.05, 212.54)	0.210
Month 11	1190.24 (288.14)	1118.93 (283.21)	94.34 (–35.45, 224.14)	0.154
**Carbohydrate, g**				
Month 07	142.45 (40.53)	127.44 (36.36)	18.26 (–0.25, 36.76)	0.053
Month 08	138.50 (38.12)	144.65 (56.59)	–2.90 (–21.41, 15.61)	0.758
Month 10	138.25 (45.72)	123.41 (41.01)	18.09 (–0.42, 36.59)	0.055
Month 11	145.81 (41.01)	126.61 (43.06)	22.45 (3.94, 40.96)	**0.018**
**Total fat, g**				
Month 07	38.49 (13.70)	43.91 (11.34)	–4.69 (–11.04, 1.65)	0.146
Month 08	37.76 (14.79)	46.21 (16.22)	–7.72 (–14.07, –1.37)	**0.017**
Month 10	39.53 (17.61)	40.34 (13.50)	–0.08 (–6.42, 6.27)	0.981
Month 11	43.99 (15.81)	43.75 (13.59)	0.97 (–5.38, 7.32)	0.764
**Saturated fat, g**				
Month 07	18.98 (7.37)	21.16 (5.98)	–1.96 (–5.27, 1.34)	0.243
Month 08	18.11 (7.41)	22.16 (8.16)	–3.83 (–7.14, –0.53)	**0.023**
Month 10	19.46 (8.88)	19.73 (7.77)	–0.05 (–3.35, 3.26)	0.977
Month 11	20.93 (8.11)	21.00 (6.84)	0.15 (–3.16, 3.45)	0.930
**NMES, g**				
Month 07	45.46 (18.22)	42.02 (17.88)	4.33 (–5.27, 13.93)	0.375
Month 08	45.90 (19.00)	49.01 (30.16)	–2.22 (–11.83, 7.38)	0.649
Month 10	40.13 (23.17)	39.00 (19.24)	2.03 (–7.58, 11.63)	0.678
Month 11	48.53 (25.02)	39.29 (21.47)	10.14 (0.54, 19.74)	**0.039**
**Sodium, mg**				
Month 07	1174.83 (656.71)	1113.54 (477.98)	84.96 (–186.02, 355.93)	0.537
Month 08	1169.36 (582.85)	1445.92 (688.80)	–252.89 (–523.87, 18.09)	0.067
Month 10	1205.99 (683.53)	1133.67 (708.06)	95.98 (–175.00, 366.96)	0.486
Month 11	1280.76 (497.31)	1271.69 (658.78)	32.74 (–238.24, 303.71)	0.812
**Protein, g**				
Month 07	46.07 (17.15)	50.13 (10.13)	–3.26 (–9.65, 3.13)	0.316
Month 08	46.09 (14.01)	56.47 (17.08)	–9.58 (–15.97, –3.19)	**0.004**
Month 10	46.45 (18.34)	44.33 (12.51)	2.92 (–3.47, 9.31)	0.369
Month 11	44.59 (14.36)	47.48 (13.13)	–2.09 (–8.48, 4.30)	0.520
**PUFA, g**				
Month 07	3.69 (1.68)	4.30 (2.06)	–0.44 (–1.48, 0.60)	0.404
Month 08	4.30 (2.29)	4.72 (3.92)	–0.26 (–1.29, 0.78)	0.623

Month 10	4.07 (1.69)	3.87 (2.17)	0.37 (–0.67, 1.40)	0.484
Month 11	5.10 (2.43)	4.34 (2.29)	0.92 (–0.11, 1.96)	0.080
**Vitamin A, μg**				
Month 07	600.40 (400.47)	571.78 (267.69)	15.37 (–106.56, 137.30)	0.804
Month 08	498.73 (321.17)	513.25 (245.67)	–27.76 (–149.70, 94.17)	0.654
Month 10	481.32 (237.81)	479.52 (263.98)	–11.45 (–133.38, 110.48)	0.853
Month 11	444.69 (236.56)	485.73 (235.35)	–54.28 (–176.21, 67.65)	0.381
**Thiamin, mg**				
Month 07	1.50 (0.63)	1.19 (0.84)	0.34 (0.03, 0.64)	**0.030**
Month 08	1.54 (0.56)	1.29 (0.72)	0.28 (–0.02, 0.59)	0.069
Month 10	1.35 (0.70)	1.03 (0.82)	0.36 (0.05, 0.66)	**0.022**
Month 11	1.36 (0.68)	0.99 (0.64)	0.40 (0.10, 0.71)	**0.010**
**Riboflavin, mg**				
Month 07	1.82 (0.64)	2.12 (0.64)	–0.29 (–0.56, –0.02)	**0.037**
Month 08	1.71 (0.54)	2.30 (0.77)	–0.58 (–0.85, –0.30)	**<0.0001**
Month 10	1.66 (0.50)	2.07 (0.67)	–0.39 (–0.66, –0.11)	**0.006**
Month 11	1.63 (0.61)	2.11 (0.57)	–0.47 (–0.74, –0.20)	**0.001**
**Niacin, mg**				
Month 07	19.97 (7.25)	17.79 (4.64)	2.49 (–0.12, 5.09)	0.061
Month 08	20.63 (5.18)	20.09 (7.30)	0.85 (–1.75, 3.45)	0.521
Month 10	19.34 (6.64)	15.80 (5.87)	3.84 (1.24, 6.45)	**0.004**
Month 11	19.09 (5.31)	17.28 (5.39)	2.11 (–0.49, 4.71)	0.112
**Vitamin B6, mg**				
Month 07	0.99 (0.57)	1.07 (0.54)	–0.07 (–0.30, 0.15)	0.524
Month 08	1.05 (0.47)	1.28 (0.79)	–0.22 (–0.44, 0.01)	0.059
Month 10	0.89 (0.43)	0.98 (0.43)	–0.07 (–0.30, 0.15)	0.514
Month 11	0.96 (0.39)	1.01 (0.38)	–0.04 (–0.26, 0.18)	0.724
**Folate, μg**				
Month 07	232.46 (113.43)	242.49 (143.98)	–6.57 (–68.96, 55.82)	0.836
Month 08	259.78 (166.42)	274.67 (134.44)	–11.44 (–73.83, 50.95)	0.718
Month 10	204.29 (144.77)	257.42 (171.64)	–49.68 (–112.07, 12.71)	0.118
Month 11	240.27 (164.95)	224.85 (87.87)	18.87 (–43.52, 81.26)	0.552
**Vitamin B12, μg**				
Month 07	2.36 (1.12)	2.78 (1.09)	–0.41 (–0.91, 0.09)	0.108
Month 08	2.25 (1.30)	3.17 (1.55)	–0.91 (–1.41, –0.41)	**0.0004**
Month 10	2.14 (0.94)	2.57 (0.93)	–0.42 (–0.92, 0.07)	0.095
Month 11	2.03 (0.91)	2.58 (1.15)	–0.55 (–1.05, –0.05)	**0.031**
**Vitamin C, mg**				
Month 07	104.00 (44.39)	45.38 (37.36)	57.38 (35.76, 79.01)	**<0.0001**
Month 08	99.95 (39.44)	50.22 (54.71)	48.48 (26.86, 70.11)	**<0.0001**
Month 10	92.84 (34.62)	50.54 (65.97)	41.05 (19.43, 62.68)	**0.0002**
Month 11	92.51 (48.54)	58.80 (61.49)	32.47 (10.85, 54.10)	**0.003**
**Vitamin D, μg**				
Month 07	6.02 (6.57)	3.23 (3.18)	2.80 (1.07, 4.53)	**0.002**
Month 08	4.73 (2.70)	3.59 (4.00)	1.16 (–0.57, 2.89)	0.188
Month 10	5.17 (2.76)	2.92 (2.49)	2.27 (0.54, 4.00)	**0.011**
Month 11	4.86 (3.44)	3.73 (4.75)	1.15 (–0.58, 2.88)	0.194
**Calcium, mg**				

Month 07	901.26 (268.15)	898.06 (287.37)	8.15 (–113.76, 130.06)	0.895
Month 08	808.31 (257.94)	943.49 (314.09)	–130.24 (–252.15, –8.33)	**0.036**
Month 10	899.34 (284.54)	836.03 (251.58)	68.25 (–53.65, 190.16)	0.271
Month 11	830.41 (284.29)	891.05 (280.79)	–55.70 (–177.60, 66.21)	0.369
**Magnesium, mg**				
Month 07	190.42 (59.23)	192.43 (50.27)	0.93 (–22.60, 24.46)	0.938
Month 08	186.24 (54.21)	206.19 (70.46)	–17.01 (–40.54, 6.52)	0.156
Month 10	181.27 (52.24)	169.37 (43.16)	14.84 (–8.70, 38.37)	0.216
Month 11	189.36 (55.05)	178.23 (46.71)	14.06 (–9.47, 37.60)	0.240
**Zinc (mg)**				
Month 07	6.75 (2.76)	6.11 (1.51)	0.71 (–0.22, 1.64)	0.133
Month 08	6.64 (2.24)	6.85 (2.65)	–0.13 (–1.07, 0.80)	0.776
Month 10	6.44 (2.45)	5.37 (1.74)	1.14 (0.21, 2.07)	**0.017**
Month 11	6.42 (1.77)	5.45 (1.69)	1.04 (0.11, 1.97)	**0.029**
**Phosphorus, mg**				
Month 07	1023.06 (284.32)	1004.05 (202.02)	33.99 (–83.88, 151.87)	0.571
Month 08	966.96 (257.85)	1106.43 (293.65)	–124.49 (–242.36, –6.62)	**0.039**
Month 10	984.67 (335.64)	930.98 (252.54)	68.68 (–49.20, 186.55)	0.252
Month 11	989.53 (278.71)	988.18 (262.19)	16.33 (–101.54, 134.20)	0.785
**Potassium, mg**				
Month 07	1666.69 (703.04)	1962.08 (433.28)	–283.10 (–528.37, –37.83)	**0.024**
Month 08	1537.51 (481.42)	2232.75 (761.17)	–682.95 (–928.21, –437.68)	**<0.0001**
Month 10	1406.79 (493.64)	1861.05 (503.26)	–441.97 (–687.24, –196.70)	**0.001**
Month 11	1512.12 (526.22)	1987.09 (511.40)	–462.67 (–707.94, –217.40)	**0.000**
**Iron, mg**				
Month 07	10.62 (3.36)	6.23 (2.82)	4.58 (3.31, 5.85)	**<0.0001**
Month 08	10.80 (2.96)	6.90 (2.75)	4.10 (2.83, 5.37)	**<0.0001**
Month 10	9.83 (2.89)	5.64 (3.07)	4.38 (3.11, 5.65)	**<0.0001**
Month 11	10.26 (3.24)	5.24 (2.35)	5.21 (3.93, 6.48)	**<0.0001**
**Copper, mg**				
Month 07	0.62 (0.32)	0.60 (0.24)	0.04 (–0.08, 0.15)	0.524
Month 08	0.60 (0.26)	0.68 (0.35)	–0.06 (–0.18, 0.05)	0.255
Month 10	0.63 (0.28)	0.50 (0.21)	0.15 (0.04, 0.26)	**0.010**
Month 11	0.60 (0.18)	0.53 (0.18)	0.09 (–0.02, 0.20)	0.115
**Selenium, µg**				
Month 07	23.53 (13.95)	26.54 (14.20)	–1.96 (–10.66, 6.75)	0.658
Month 08	25.49 (21.96)	34.94 (36.19)	–8.39 (–17.09, 0.31)	0.059
Month 10	25.85 (17.66)	22.68 (13.11)	4.22 (–4.48, 12.92)	0.340
Month 11	25.66 (19.68)	25.02 (13.39)	1.70 (–7.00, 10.40)	0.701
**Iodine, µg**				
Month 07	64.08 (23.15)	52.65 (24.78)	11.80 (0.49, 23.11)	**0.041**
Month 08	63.58 (29.84)	55.72 (21.88)	8.22 (–3.09, 19.54)	0.154
Month 10	60.53 (27.92)	53.13 (26.95)	7.77 (–3.55, 19.08)	0.178
Month 11	65.92 (30.56)	53.53 (21.06)	12.76 (1.45, 24.07)	**0.027**

EI: Energy intake; N/A: Not available.
* Significant *p*–values are bold.
[1] Repeated measures mixed model with an autoregressive covariance structure, adjusting for sex.
[2] Only nutrients with significant relationships at any of the four time points are displayed.
